# Supplementary material for: Analysis of DIA proteomics data using MSFragger-DIA and FragPipe computational platform
Source: Nat Commun. 2023 Jul 12;14:4154. doi: 10.1038/s41467-023-39869-5 (PMC10338508; doi:10.1038/s41467-023-39869-5)
Supplement: Supplementary file 1 — Supplementary Information [file 41467_2023_39869_MOESM1_ESM.pdf]

Supplement to: "Analysis of DIA proteomics data using MSFragger-DIA and FragPipe computational platform"

Fengchao Yu[1]*, Guo Ci Teo[1], Andy T. Kong[1,2], Klemens E. Fröhlich[3], Ginny Xiaohe Li[1], Vadim Demichev[4,5], Alexey I. Nesvizhskii[1,2]*

[1]Department of Pathology, University of Michigan, Ann Arbor, Michigan, USA

[2]Department of Computational Medicine and Bioinformatics, University of Michigan, Ann Arbor, Michigan, USA

[3]Proteomics Core Facility, Biozentrum, University of Basel, Basel, Switzerland

[4]Department of Biochemistry, Charité Universitätsmedizin Berlin, Germany

[5]Department of Biochemistry, University of Cambridge, Cambridge, UK

* Correspondence to F.Y. (yufe@umich.edu) and A.I.N. (nesvi@med.umich.edu)

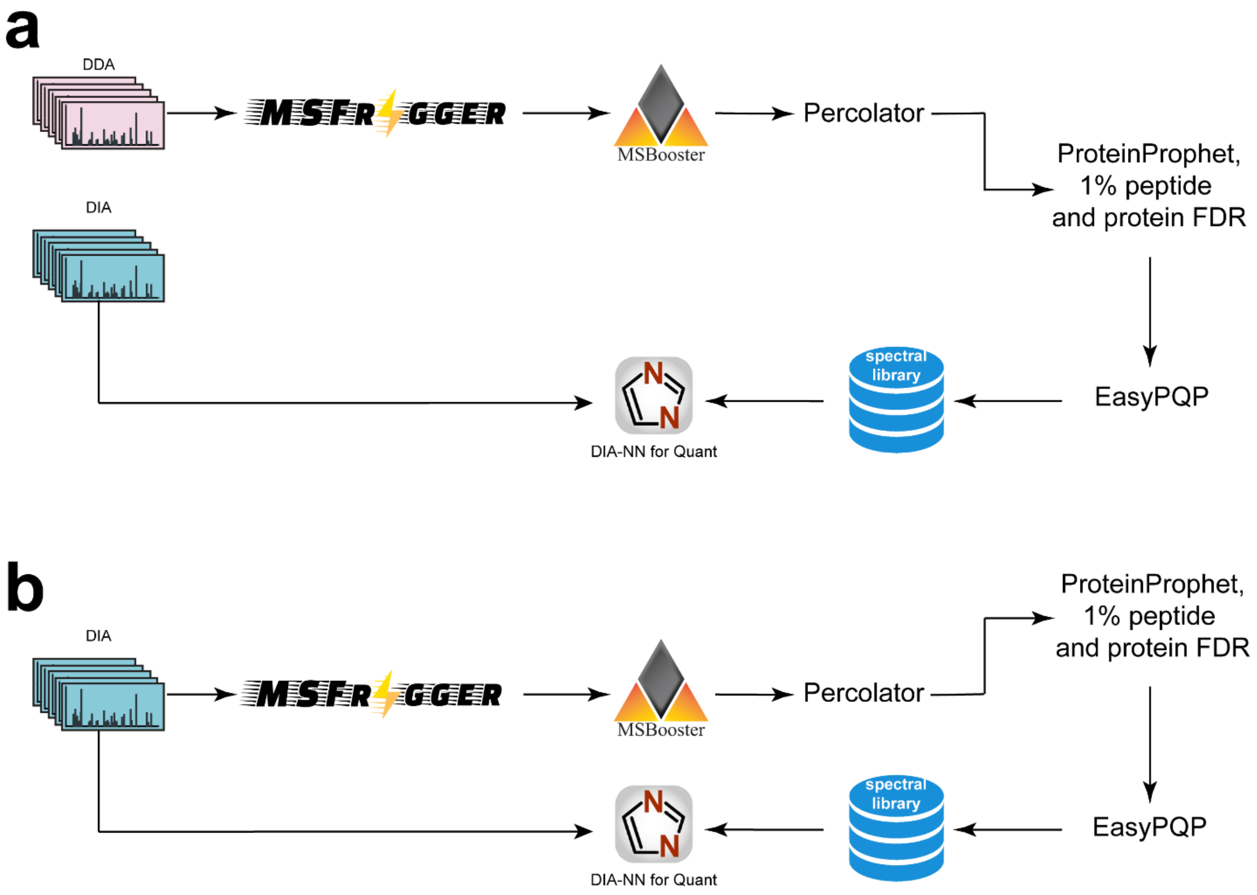

Supplementary Figure 1. DIA data analysis pipelines covering library-based, library-free, and hybrid approaches. **(a) Library-based analysis.** MSFragger in DDA mode is used to search the DDA data, followed by MSBooster, Percolator, ProteinProphet, FDR filtering, and spectral library generation with EasyPQP. DIA-NN is used to quantify DIA data using the spectral library from the DDA data. It is library-based because it requires DDA runs as a library. **(b) Library-free analysis.** MSFragger-DIA is used to search the DIA data to build a spectral library. Then, the spectral library is used to quantify the DIA data. It is library-free because it does not require DDA runs or pre-generated library.

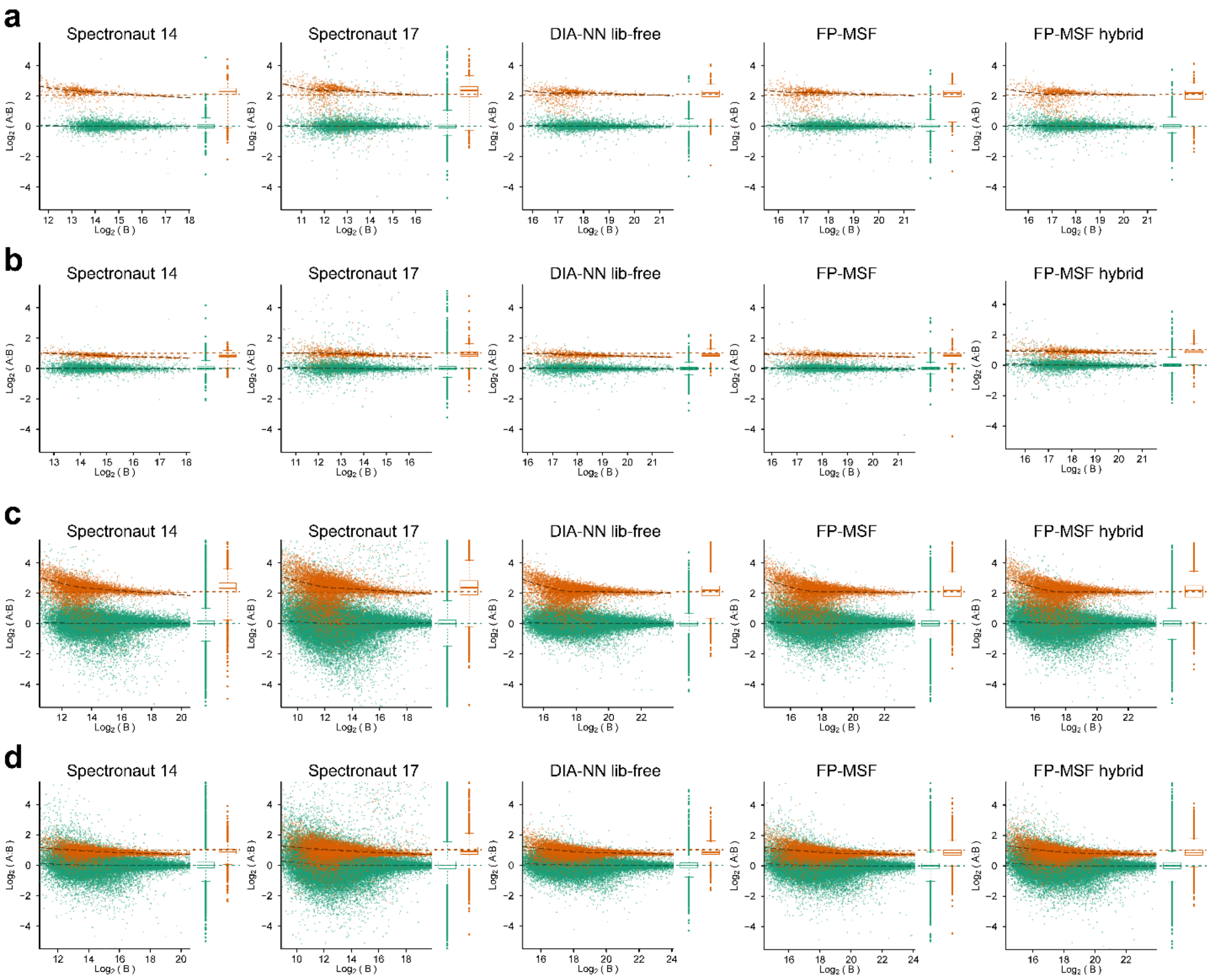

Supplementary Figure 2. Quantification accuracy evaluation using the benchmark dataset. **(a)** Scatter plots depicting the relationship between protein log2 ratio and intensity, using proteins from the "1-06" (condition A) and "1-25" (condition B) conditions to compute log-ratios. There are 2 conditions. Each condition contains 23 replicates. E. coli proteins are colored orange, while H. sapiens proteins appear in green. Horizontal dashed lines indicate true log-ratios, while adjacent box plots display the marginal distribution of log-ratios on the right side of each scatter plot. The box in each box plot captures the interquartile range (IQR), with the bottom and top edges representing the first (Q1) and third quartiles (Q3) respectively. The median (Q2) is marked by a horizontal line within the box. The whiskers extend to the minima and maxima within 1.5 times the IQR below Q1 or above Q3. Outliers, signified by individual dots, fall outside these bounds. **(b)** Same as (a) for the proteins from the “1-06” (condition A) and “1-12” (condition B). **(c)** Same as (a) for the precursor-level results. **(d)** Same as (b) for the precursor-level results.

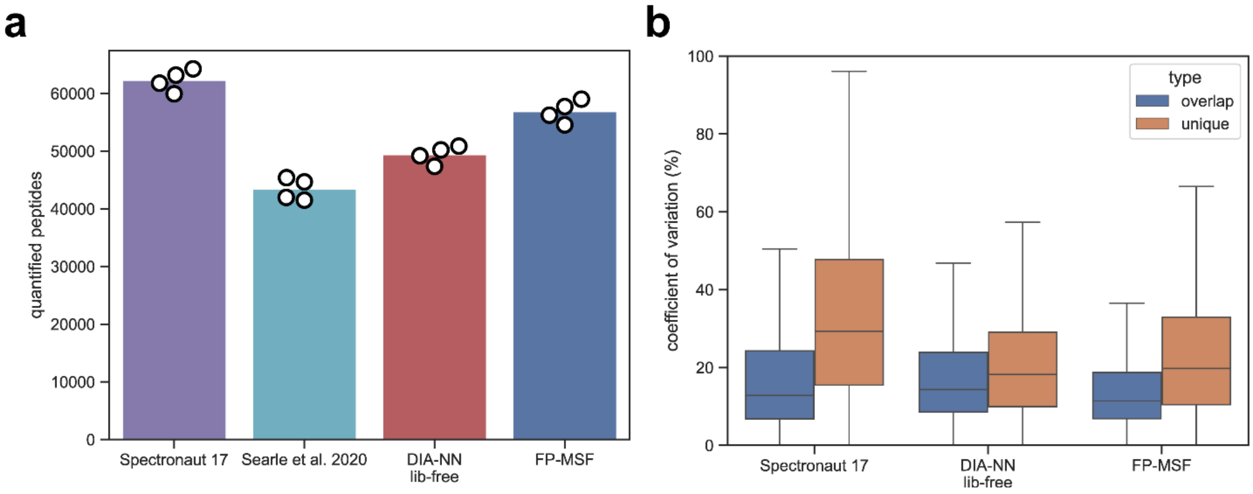

Supplementary Figure 3. Quantified peptides and coefficient of variation (CV) from the 2020-Yeast dataset. **(a)** Bar plots of the quantified peptides. The bar height is the average number of four replicates. The white dots indicate the numbers from individual replicates. The results from the original publication (obtained using EncyclopeDIA version 0.8.3) are shown. The latest EncyclopeDIA (version 2.12.30) crashed with errors. **(b)** Box plots of peptide CVs. There are 4 single-shot DIA runs from 4 replicates. Blue box plots represent overlapping peptides shared among all tools, while brown box plots depict unique peptides quantified exclusively by each specific tool. The box in each box plot captures the IQR, with the bottom and top edges representing the Q1 and Q3 respectively. The median is marked by a horizontal line within the box. The whiskers extend to the minima and maxima within 1.5 times the IQR below Q1 or above Q3.

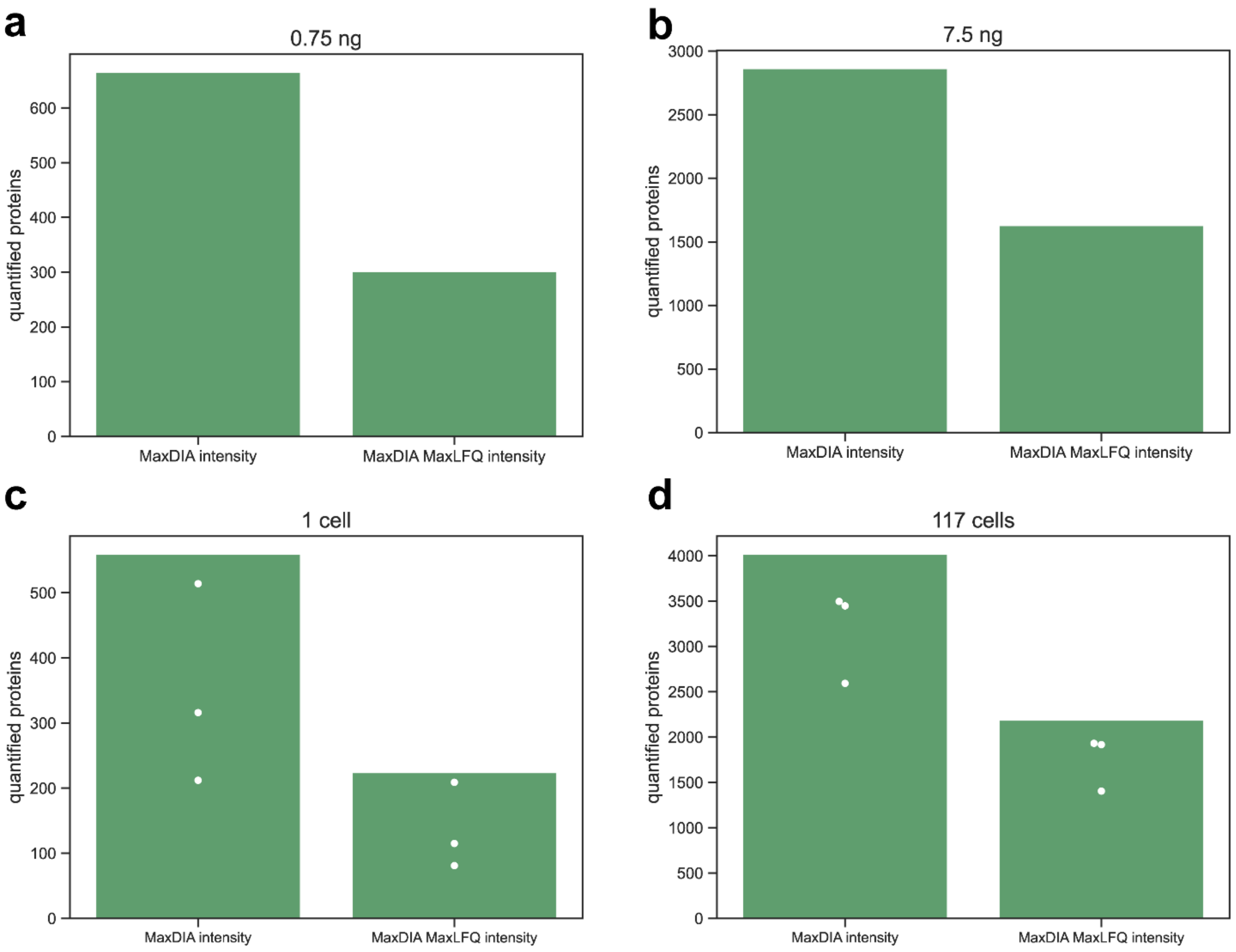

Supplementary Figure 4. Quantified proteins from the low-input-cell and the single-cell datasets using MaxDIA. The original intensity and the MaxLFQ intensity were used to count the quantified proteins. **(a-b)** Bar plots from analyzing the low-input-cell dataset. Proteins with zero intensities are discarded. **(c-d)** Bar plots from analyzing the single-cell datasets. The bar height is the total number of proteins from the three replicates. The white dots are the numbers of proteins quantified in each replicate.

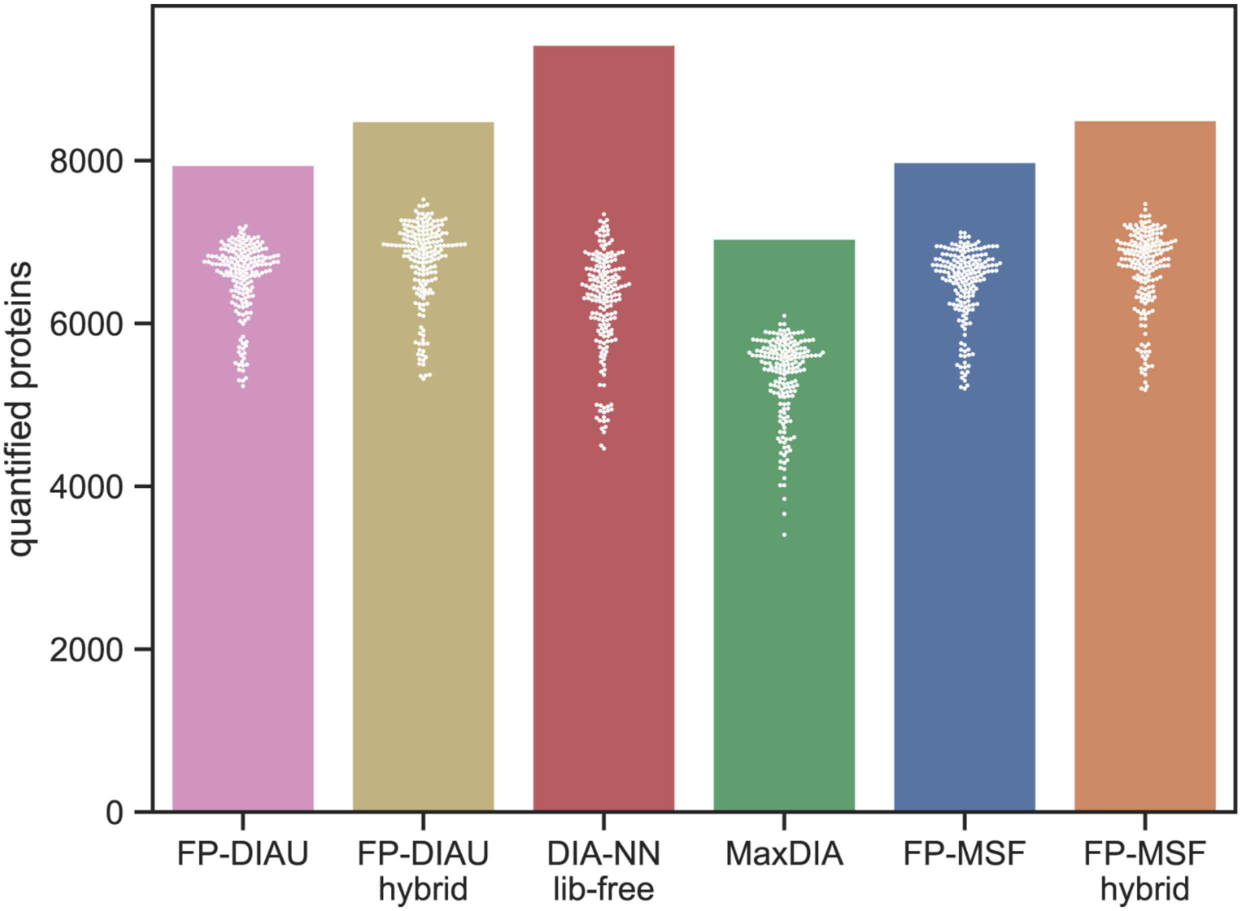

Supplementary Figure 5. Bar plots of the number of quantified proteins in the ccRCC dataset. There are 187 independent biological samples. The bar height is the total number. The white dots are the numbers from individual runs. Proteins with at least one non-zero intensity are included.
