# Supplementary material for: Analysis of DIA proteomics data using MSFragger-DIA and FragPipe computational platform
Source: Nat Commun. 2023 Jul 12;14:4154. doi: 10.1038/s41467-023-39869-5 (PMC10338508; doi:10.1038/s41467-023-39869-5)
Supplement: Supplementary file 3 — Description of Additional Supplementary Files [file 41467_2023_39869_MOESM3_ESM.pdf]

Title: Supplementary Data 1

Description: Mass spectrometry data files used in this study. The first column is the dataset name used in the manuscript. The second column is the data identifier in ProteomeXchange, European Genome-phenome Archive, or National Cancer Institute Proteomic Data Commons. The third column is the run names of each file. The fourth column is the corresponding figures in the manuscript. The fifth column is the instrument used to collect the data. The sixth column is the acquisition scheme. The seventh column is the gradient length.

Title: Supplementary Data 2

Description: Numbers of *E. coli* precursors, *H. Sapiens* precursors, and the estimated actual FDR from the benchmark dataset. Tools, including Spectronaut, DIA-NN, and FragPipe coupled with MSFragger-DIA (FP-MSF), were used. FP-MSF with and without experimental spectra were tested. Precursors assigned to both *E. coli* and *H. Sapiens* were discarded to avoid ambiguity.

Title: Supplementary Data 3

Description: OmicsEV output for the ccRCC dataset.
