# Supplementary material for: Analysis of DIA proteomics data using MSFragger-DIA and FragPipe computational platform
Source: Nat Commun. 2023 Jul 12;14:4154. doi: 10.1038/s41467-023-39869-5 (PMC10338508; doi:10.1038/s41467-023-39869-5)
Supplement: Supplementary file 7 — Reporting Summary [file 41467_2023_39869_MOESM7_ESM.pdf]

nature portfolio

Corresponding author(s): Fengchao Yu, Alexey I. Nesvizhskii

Last updated by author(s): Jun 12, 2023

Reporting Summary

Nature Portfolio wishes to improve the reproducibility of the work that we publish. This form provides structure for consistency and transparency in reporting. For further information on Nature Portfolio policies, see our Editorial Policies and the Editorial Policy Checklist.

Statistics

For all statistical analyses, confirm that the following items are present in the figure legend, table legend, main text, or Methods section.

n/a	Confirmed	
☐	☒	The exact sample size (*n*) for each experimental group/condition, given as a discrete number and unit of measurement
☒	☐	A statement on whether measurements were taken from distinct samples or whether the same sample was measured repeatedly
☐	☒	The statistical test(s) used AND whether they are one- or two-sided<br>*Only common tests should be described solely by name; describe more complex techniques in the Methods section.*
☒	☐	A description of all covariates tested
☒	☐	A description of any assumptions or corrections, such as tests of normality and adjustment for multiple comparisons
☒	☐	A full description of the statistical parameters including central tendency (e.g. means) or other basic estimates (e.g. regression coefficient) AND variation (e.g. standard deviation) or associated estimates of uncertainty (e.g. confidence intervals)
☒	☐	For null hypothesis testing, the test statistic (e.g. *F*, *t*, *r*) with confidence intervals, effect sizes, degrees of freedom and *P* value noted<br>*Give P values as exact values whenever suitable.*
☒	☐	For Bayesian analysis, information on the choice of priors and Markov chain Monte Carlo settings
☒	☐	For hierarchical and complex designs, identification of the appropriate level for tests and full reporting of outcomes
☒	☐	Estimates of effect sizes (e.g. Cohen's *d*, Pearson's *r*), indicating how they were calculated

Our web collection on statistics for biologists contains articles on many of the points above.

Software and code

Policy information about availability of computer code

Data collection	No data was collected. All data used in the experiments was from public repositories.
Data analysis	Software used in this study: FragPipe v18.0, MSFragger v3.5, Philosopher, DIA-NN v1.8.1, MaxQuant v2.2.0.0, MaxQuant v2.1.3.0, ProteoWizard, Spectronaut v14, Spectronaut v17, OmicsEV, Python, R, EncyclopeDIA v2.12.30, custom scripts in https://github.com/Nesvilab/MSFragger-DIA-manuscript

For manuscripts utilizing custom algorithms or software that are central to the research but not yet described in published literature, software must be made available to editors and reviewers. We strongly encourage code deposition in a community repository (e.g. GitHub). See the Nature Portfolio guidelines for submitting code & software for further information.

Data

Policy information about availability of data

All manuscripts must include a data availability statement. This statement should provide the following information, where applicable:

- Accession codes, unique identifiers, or web links for publicly available datasets
- A description of any restrictions on data availability
- For clinical datasets or third party data, please ensure that the statement adheres to our policy

No new data was collected. The parameter, log, and result files from all tools used in this manuscript can be found from https://doi.org/10.5281/zenodo.7261712. The raw mass spectrometry data were downloaded from the MassIVE (MSV000082805, MSV000084000), ProteomeXchange (PXD022992, PXD023325, and

PXD027679), National Cancer Institute Proteomic Data Commons (PDC000200), and European Genome-phenome Archive (EGAD00010002223). The proteome database files were downloaded from the UniProt with proteome ID: UP000005640 (H. sapiens), UP000002311 (S. cerevisiae), UP000000625 (E. coli), and UP000006548 (A. thaliana). The contaminant proteins were downloaded from https://www.thegpm.org/crap/.

Human research participants

Policy information about studies involving human research participants and Sex and Gender in Research.

Reporting on sex and gender	NA
Population characteristics	NA
Recruitment	NA
Ethics oversight	NA

Note that full information on the approval of the study protocol must also be provided in the manuscript.

Field-specific reporting

Please select the one below that is the best fit for your research. If you are not sure, read the appropriate sections before making your selection.

☒ Life sciences ☐ Behavioural & social sciences ☐ Ecological, evolutionary & environmental sciences

For a reference copy of the document with all sections, see nature.com/documents/nr-reporting-summary-flat.pdf

Life sciences study design

All studies must disclose on these points even when the disclosure is negative.

Sample size	This study used the LC-MS data from the public repositories, with sample sizes determined by the authors of the original publications. We employed seven public datasets to assess our tools' performance. The datasets are as follows: the first comprises 92 independent samples; the second, one sample with three replicates; the third, a single sample with four replicates; the fourth and fifth, two samples each with three replicates; the sixth, six samples each with two replicates; and the seventh, 187 independent samples.
Data exclusions	No data was excluded.
Replication	Seven large-scale datasets from different acquisition protocols and studies were used to evaluate the performance of our tools. All data is from the public repositories. Each dataset has biological or technical replicates to evaluate the reproducibility, precision, and accuracy. All benchmarking tools analyzed the same set of data to ensure the fair comparison.
Randomization	The public LC-MS data was grouped based on the original publications' studies. Each tool analyzed the data in these groupings to ensure fair comparison. Data from different studies was not mixed.
Blinding	Blinding is not relevant because this study focuses on software development. All benchmarking tools used the same set of data to have fair comparisons.

Reporting for specific materials, systems and methods

We require information from authors about some types of materials, experimental systems and methods used in many studies. Here, indicate whether each material, system or method listed is relevant to your study. If you are not sure if a list item applies to your research, read the appropriate section before selecting a response.

Materials & experimental systems

n/a	Involved in the study	
☒	☐	Antibodies
☒	☐	Eukaryotic cell lines
☒	☐	Palaeontology and archaeology
☒	☐	Animals and other organisms
☒	☐	Clinical data
☒	☐	Dual use research of concern

Methods

n/a	Involved in the study	
☒	☐	ChIP-seq
☒	☐	Flow cytometry
☒	☐	MRI-based neuroimaging
